# Clinical utility of brain-derived neurotrophic factor as a biomarker with left ventricular echocardiographic indices for potential diagnosis of coronary artery disease

**DOI:** 10.1038/s41598-020-73296-6

**Published:** 2020-10-01

**Authors:** K. G. Monisha, Paramasivam Prabu, M. Chokkalingam, Ram Murugesan, Dragan Milenkovic, Shiek S. S. J. Ahmed

**Affiliations:** 1 Department of Cardiology, Chettinad Hospital and Research Institute, Chettinad Health City, Kelambakkam, Tamil Nadu 603103 India; 2 School of Medicine, Department of Neurology, University of New Mexico Health Sciences Center, University of New Mexico, Albuquerque, USA; 3 Drug Discovery and Multi-Omics Laboratory, Faculty of Allied Health Sciences, Chettinad Academy of Research and Education, Kelambakkam, Tamil Nadu 603103 India; 4 Université Clermont Auvergne, INRAe, UNH, Clermont-Ferrand, France

**Keywords:** Molecular biology, Biomarkers, Cardiology, Mathematics and computing

## Abstract

Brain-derived neurotrophic factor (BDNF) plays a central pivotal role in the development of the cardiovascular system. Recent evidence suggests that BDNF has adverse subclinical cardiac remodeling in participants with cardiovascular disease risk factors. Relating serum BDNF levels with two-dimensional echocardiographic indices will provide insights into the BDNF mediated pathophysiology in coronary artery disease (CAD) that may shed light upon potential diagnostic biomarkers. For the study, 221 participants were recruited and classified based on coronary angiogram examination as control (n = 105) and CAD (n = 116). All participants underwent routine blood investigation, two-dimensional echocardiography, and serum BDNF estimation. As a result, total cholesterol, triglyceride, low-density lipid, high-density lipid, HbA1c (glycosylated hemoglobin), serum creatinine, eosinophils, lymphocyte, monocytes, neutrophils, and platelets were significantly elevated in CAD individuals compared to controls. Notably, the serum BDNF was significantly lower in individuals with CAD (30.69 ± 5.45 ng/ml) than controls (46.58 ± 7.95 ng/ml). Multivariate regression analysis showed neutrophils, total cholesterol, left ventricular mass index, mitral inflow E/A ratio, and pulmonary vein AR duration were associated with low BDNF in CAD. Four independent support vector machine (SVM) models performed to ensure the BDNF level in the classification of CAD from healthy controls. Particularly, the model with serum BDNF concentration and blood parameters of CAD achieved significant improvement from 90.95 to 98.19% in detecting CAD from healthy controls. Overall, our analysis provides a significant molecular linkage between the serum BDNF level and cardiovascular function. Our results contribute to the emerging evidence of BDNF as a potential diagnostic value in CAD that might lead to clinical application.

## Introduction

Coronary artery disease (CAD) is one of the significant causes of mortality worldwide. Endothelial dysfunction, inflammation, and atherosclerotic plaque formation lead to CAD^1^. Such events accelerated by diabetes, hypertension, smoking, and obesity^2^. Although these risk factors predict cardiovascular events, the molecules and their mechanism involved in CAD development are still under investigation. In the modern era, several molecular techniques such as genomics, transcriptomics, proteomics, and metabolomics plays a crucial role to understand the molecular mechanism of CAD, which pave the way to discover molecular diagnostic markers^3^.

In recent times several markers such as high sensitive c-reactive protein (hs-CRP), interleukins, monocyte chemoattractant protein-1 (MCP-1), TNF-α, serum amyloid A (SAA), vascular adhesion molecule 1 (VCAM-1), selectins, vascular endothelial growth factor (VEGF), matrix metalloproteinases (MMPS) and soluble CD40 ligand is identified^4^. Of which, few are clinically useful to detect CAD, whereas other markers are neglected due to lack of direct involvement pathophysiological events of CAD^5^. For example, hs-CRP is one of the known inflammatory markers proven associated with two-dimensional echocardiography of CAD individuals^6^. Therefore, any new marker needs to be cross-linked with the gold standard imaging techniques making it useful for clinical application.

Brain-derived neurotrophic factor (BDNF) is a neurotrophic protein expressed in the central and peripheral circulation^7^. Although BDNF is a neurotrophic protein, it plays a vital role in the cardiac system^8^. Importantly, BDNF involved in the development of heart^9^, endothelial cells^10^, vascular smooth muscle cells^11^, macrophages^12^, lymphocytes^13^ and atherosclerotic vessels^11^. Recent research emphasizes that BDNF induces oxidative stress by activating oxidase enzyme in the coronary artery vasculature^14^. Low serum BDNF was observed in patients with CAD that causing future coronary events^15^. In general, the ventricular function has been assessed by the two-dimensional echocardiographic imaging recommended by the American Society of Echocardiography (ASE)^16^. Relating serum BDNF levels with echocardiographic indices may provide the involvement of BDNF in cardiac systems, which may be useful in developing a potential alternative method for CAD diagnosis. Recently Bahls et al., 2019 showed the association between low BDNF and echocardiographic indices in participants with traditional risk factors of future CAD^17^. However, the role of BDNF with cardiac left ventricular function and its ability to classify CAD from healthy controls is less well known.

This study aimed to investigate the relationship between serum BDNF levels with biochemical and echocardiographic indices in 221 participants for its utility as a CAD marker. We identified a significant molecular linkage between the BDNF with biochemical changes and echocardiographic indices associated with cardiovascular function. Further, we construct an automated CAD detection model using a support vector machine with BDNF showing improved accuracy making a rapid decision in CAD.

## Results

### Patients characteristics and comparative analysis

The clinical and anthropometric characteristics of the participants involved in this study was illustrated in Table 1. All descriptive data expressed as mean ± standard deviation for control and CAD. All participants in control group are free from risk factors, Whereas the CAD group contains 10.2% of hypertensive, 15.3% of diabetes mellitus and 5.5% of smokers. However, most CAD participants in CAD are free from the risk factors. Further, the statistical t-test analysis showed a significant change in BMI, platelet, eosinophils, lymphocyte, monocytes, neutrophils, HbA1c (glycosylated hemoglobin), serum creatinine, low density lipid (LDL), high density lipid (HDL), triglyceride (TGL), and total cholesterol in CAD compared to control (Table 1). Mainly, serum BDNF was significantly (*p* ≤ 0.001) decreased in CAD (30.70 ± 5.4 ng/ml) compared to the control (46.580 ± 7.9 ng/ml). Although basophils, systolic, and diastolic pressure showed variations in CAD, but not statistically significant.

**Table 1:** Characteristics of the study population in anthropometric and blood parameters.

Clinical parameters	Without CAD	With CAD	*p* value
Age (years)	54.49 ± 11.40	57.25 ± 10.44	0.062
BMI (kg/m^2^)	22.64 ± 2.27	26.23 ± 3.05	< 0.001*
Systolic BP (mmHg)	121.37 ± 13.60	123.70 ± 17.40	0.271
Diastolic BP (mmHg)	81.61 ± 12.52	82.99 ± 13.18	0.426
Platelet (LAC/C.mm)	2.18 ± 0.77	2.91 ± 1.05	< 0.001*
Basophil (%)	0.82 ± 0.43	0.88 ± 0.66	0.4
Eosinophil (%)	5.06 ± 2.20	6.53 ± 3.13	< 0.001*
Lymphocyte (%)	29.38 ± 7.73	36.27 ± 14.59	< 0.001*
Monocyte (%)	5.66 ± 2.02	7.85 ± 2.59	< 0.001*
Neutrophil (%)	52.24 ± 12.67	57.89 ± 17.71	< 0.001*
HbA1c (%)	4.73 ± 0.38	6.42 ± 1.16	< 0.001*
HDL cholesterol (mg/dL)	47.12 ± 9.00	35.81 ± 8.24	< 0.001*
LDL cholesterol (mg/dL)	84.27 ± 15.07	132.85 ± 36.95	< 0.001*
TGL cholesterol (mg/dL)	96.29 ± 18.43	142.16 ± 59.04	< 0.001*
T.Cholesterol (mg/dL)	131.25 ± 24.77	194.69 ± 66.03	< 0.001*
Serum creatinine (mg/dL)	0.84 ± 0.23	1.06 ± 0.40	< 0.001*
BDNF (ng/mL)	46.58 ± 7.95	30.69 ± 5.45	< 0.001*

*Significance at *p* < 0.05.

### Assessment of echocardiography indices

Next, we assessed the echocardiographic indices in 221 participants. Increased left atrial diameter (LAD), left ventricular mass index (LVMI), isovolumetric relaxation time (IVRT), pulmonary vein systole / diastole ratio (PV S/D) and decreased biplane left ventricular ejection fraction (LVEF), Mitral inflow E/A ratio (MV E/A ratio), mitral septal peak myocardial early diastolic velocity (S E/e′ ratio), mitral lateral peak myocardial early diastolic velocity (L E/e′ ratio), pulmonary vein AR duration (PV AR), and global longitudinal strain (GLS LVEF) were observed in the CAD compared to the control. The statistical analysis confirms the variation in echocardiographic indices of CAD compared to control (Table 2).

**Table 2:** Echocardiographic indices of the participants with CAD and without CAD. Biplane LVEF (biplane left ventricular ejection fraction); LAD (left atrial diameter), LVMI (left ventricular mass index), MV E/A (Mitral inflow E/A ratio), Le’ /Se’(mitral lateral/septal peak myocardial early diastolic velocity), IVRT (isovolumetric relaxation time), PV AR (pulmonary vein AR duration), PV S/D (pulmonary vein systole / diastole ratio) and GLS EF (global longitudinal strain).

Echo indices	Without CAD	With CAD	*p* value
Biplane LVEF (%)	61.08 ± 3.01	44.75 ± 7.01	< 0.001*
LAD (cm)	3.25 ± 0.30	3.45 ± 0.51	< 0.001*
LVMI (g/m2)	82.31 ± 24.67	100.69 ± 24.35	< 0.001*
MV E/A	1.06 ± 0.30	0.64 ± 0.45	< 0.001*
S E/e'	8.42 ± 1.01	7.20 ± 1.28	< 0.001*
L E/e'	10.46 ± 1.04	7.62 ± 1.14	< 0.001*
IVRT (ms)	80.81 ± 16.34	114.57 ± 23.61	< 0.001*
PV AR (m/s)	32.19 ± 5.80	27.78 ± 5.24	< 0.001*
PV S/D	0.69 ± 0.23	1.29 ± 0.57	< 0.001*
GLS EF (%)	− 16.18 ± 0.47	− 13.94 ± 1.27	< 0.001*

*Significance at *p* < 0.05.

### BDNF and CAD associated clinical and echocardiography parameters

To determine the association between serum BDNF levels and clinical parameters, the CAD patients were quartile grouped as low and high (described in the method section). The BDNF concentration with ≤ 29.91 ng/ml considered as a low BDNF group, and those with levels > 29.92 ng/ml are considered a high BDNF group. The Supplementary Table A1 shows the characteristics of collected blood parameters and echocardiographic indices in low and high BDNF group. Elevated platelets, basophils, eosinophils, lymphocytes, monocytes, neutrophils, LDL, TGL, total cholesterol, HbA1c, and serum creatinine were observed in low BDNF group of CAD patients. In contrast, significant decreased BMI and HDL noticed in low BDNF groups compared to the high BDNF group. Similarly, the echocardiographic indices in low BDNF shows evaluated Biplane LVEF, PV AR (m/s), GLS LVEF, and decreased LVMI, MV E/A ratio, IVRT (ms), and PV S/D ratio compared to the high BDNF group. The statistical analysis of blood parameters and echocardiographic indices confirms the significance between the low and high BDNF groups, except LAD, MV S E/e’ ratio, and L E/e’ ratio (Supplementary Table A2).

### Multivariate regression analysis

We performed a multivariate regression analysis to determine the blood parameters influencing low BDNF concentration in CAD (Table 3). The neutrophils (β =  − 0.494, *p* ≤ 0.001) and total cholesterol (β =  − 0.407, *p* ≤ 0.001) were noticed to contribute low BDNF concentration in CAD with a model fit measure of r = 0.725 and r-square = 0.526. Similarly, the echocardiographic indices (Table 4), LVMI (β = 0.3380, *p* ≤ 0.001), MV E/A (β =  − 0.3751, *p* ≤ 0.001), PV AR (β =  − 0.3444, *p* ≤ 0.001) and Biplane LVEF (β = 0.3467, *p* = 0.010) were significantly linked with low BDNF concentration in CAD with a model fit measure r = 0.826 and r-square = 0.683. Overall, the analysis suggests low BDNF concentration is the measure of both blood (neutrophils and total cholesterol) and echocardiographic indices (LVMI, MV E/A, PV AR, and Biplane LVEF) in CAD.

**Table 3:** Multivariate regression analysis showing low BDNF contributing blood parameters in CAD. Data are represented with β and *p* values with 95% confidence interval.

Predictor	Univariate analysis			Multivariate analysis		
	Coefficient correlation	*p* value	95% confidence interval	β	95% confidence interval	*p* value
Age (years)	0.06	0.63	− 0.075 to 0.04			
BMI (kg/m2)	0.11	0.38	− 0.12 to 0.30			
Systolic BP (mmHg)	0.05	0.70	− 0.04 to 0.03			
Diastolic BP (mmHg)	0.03	0.79	− 0.04 to 0.05			
Platelet (LAC/C.mm)	0.11	0.40	− 0.38 to 0.92			
Basophil (%)	0.04	0.76	− 0.754 to 1.03			
Eosinophil (%)	0.04	0.73	− 0.28 to 0.20			
Lymphocyte (%)	0.02	0.87	− 0.03 to 0.04			
Monocyte (%)	0.12	0.35	− 0.13 to 0.37			
Neutrophil (%)	0.61	< 0.001*	− 0.11 to 0.05	− 0.494	− 0.09 to 0.04	< 0.001*
HDL cholesterol (mg/dL)	0.01	0.93	− 0.08 to 0.09			
LDL cholesterol (mg/dL)	0.12	0.34	− 0.01 to 0.02			
TGL cholesterol (mg/dL)	0.01	0.91	− 0.0098 to 0.01			
T.Cholesterol (mg/dL)	0.54	< 0.001*	− 0.02 to 0.01	− 0.407	− 0.020 to 0.007	< 0.001*
HbA1c (%)	0.013	0.92	− 0.52 to 0.58			
Serum Creatinine (mg/dL)	0.12	0.3	− 1.03 to 2.72			

*Significance at *p* < 0.05.

**Table 4:** Multivariate regression analysis showing low BDNF contributing Echocardiographic indices in CAD. Data are represented with β and *p* values with 95% confidence interval.

Predictor	Univariate analysis			Multivariate analysis		
	Coefficient correlation	*p* value	95% confidence interval	β	95% confidence interval	*p* value
Biplane LVEF (%)	0.45	< 0.001*	0.09 to 0.30	0.35	0.03 to 0.26	0.01*
LAD (cm)	0.38	0.003*	0.752 to 3.5	0.13	− 0.19 to 1.69	0.117
LVMI (g/m2)	0.52	< 0.001*	0.034 to 0.09	0.33	0.01 to 0.05	< 0.001*
MV E/A	0.32	0.012*	0.80 to 6.36	− 0.37	− 2.63 to− 0.97	< 0.001*
S E/e'	0.04	0.743	− 0.86 to 1.21			
L E/e'	0.01	0.897	− 1.40 to 1.59			
IVRT (ms)	0.18	0.163	− 0.01 to 0.06			
PV AR (m/s)	0.61	< 0 .001*	0.18 to 0.38	0.34	0.06 to 0.24	< 0.001*
PV S/D	0.12	0.338	− 0.95 to 2.73			
GLS EF (%)	0.45	< 0.001*	− 1.62 to 0.497	0.05	− 0.47 to 0.71	0.686

*Indicate significance at *p* < 0*.*05*.*

### Support vector machine classification of CAD from control

Support vector machine (SVM) based classification model was developed to determine the influence of BDNF as a biomarker in predicting CAD. Prior to disease classification, we use SVM Attribute Evaluator with the ranker method to select most contributing predictor variables for CAD from clinical and echocardiographic indices. The BMI, HbA1c, HDL, LDL, and Total cholesterol were identified as the five most important blood predictor variables of CAD. Whereas in echocardiographic indices, Biplane LVEF, LAD, L E/e, IVRT, and GLS LVEF were determined as important indices for CAD prediction (Table 5). To check influence of BDNF in predicting CAD, four independent SVM models were developed, the model-A was trained with BMI, HbA1c, HDL, LDL, and Total cholesterol (Table 5). The model-B contains Biplane LVEF, LAD, L E/e, IVRT, and GLS LVEF echocardiographic indices (Table 5). The model-C was trained with model-A attributes along with BDNF concentration. Similarly, model-D was trained with model-B attributes along with BDNF concentration. The accuracy, True Positive (TP) rate, False Positive (FP) rate, precision, recall, F-measure, and Receiver operating characteristic (ROC) of each model shown in Table 5. All SVM models showed accuracy over 90%. For instance, model-A showed 90.95% accuracy, whereas adding the serum BDNF concentration in model-C showed 98.19% improved accuracy in detection CAD. As expected, the model-B with echocardiographic indices revealed high (98.64%) accuracy as the echocardiographic imaging is the important tool that routinely used in detecting CAD. However, the model-D with serum BDNF concentration as an additional attribute to the other echocardiographic parameters of model-B resulted 100% accuracy in detecting CAD.

**Table 5:** Model describing the parameters associated with the accuracy in classifying CAD from healthy control.

Model	Predictor variables	TP rate	FP rate	Precision	Recall	F-measure	ROC area	Accuracy (%)
Model-A	BMI, HbA1c, HDL, LDL, and Total cholesterol	0.911	0.086	0.913	0.910	0.91	0.912	90.95
Model-B	Biplane LVEF, LAD, L E/e, IVRT, and GLS LVEF	0.986	0.012	0.987	0.986	0.986	0.986	98.64
Model-C	BDNF, BMI, HbA1c, HDL, LDL, and Total cholesterol	0.986	0.015	0.987	0.986	0.986	0.986	98.19
Model-D	BDNF, Biplane LVEF, LAD, L E/e, IVRT, and GLS LVEF	1	0	1	1	1	1	100

*TP* true positive, *FP* false positive, *ROC* receiver operating characteristic.

## Discussion

BDNF has been extensively studied to promote neurogenesis^18^ and also plays a dominant role in the cardiovascular system^19^. BDNF expressed in endothelial cells, vascular smooth muscle cells, macrophages, lymphocytes, and atherosclerotic vessels^14^. Framingham Heart Study 2015 suggests that high BDNF concentration was associated with a decreased risk of CVD and mortality^15^. A recent study suggests the involvement of BDNF in oxidative stress in coronary artery vasculature and atherosclerotic plaque formation^20^. Despite various studies, the association of BDNF in CAD with non-invasive cardiovascular imaging techniques like two-dimension echocardiography imaging has not been reported. Therefore, we investigated the involvement of BDNF in CAD compared to healthy control. Elucidating the association of BDNF with CAD associated parameters may enable utilization of BDNF as a possible diagnostic biomarker for CAD. Particularly, establishing the relationship between alerted serum BDNF level and echocardiographic indices will help develop a potential alternative method for CAD diagnosis. This is the first study conducted in South Indian ethnicity to assess the association of serum BDNF levels with clinical parameters and echocardiography indices in CAD. We segmented our study design into three-fold. First, we confirmed the significant changes in blood parameters, imaging indices, and BDNF levels in CAD compared to controls. Second, we use multivariate regression analysis to determine the blood parameters and imaging indices influencing low BDNF concentration in CAD. Finally, we generate SVM models to determine the influence BDNF in improving the classification of CAD from control by including and excluding the BDNF attribute while training and testing the models.

Our present study showed decreased serum BDNF in CAD which is in agreement with Eyileten et al., 2016, confirming the decreased serum BDNF that correlates with VCAM1 and soluble P-selectin in CAD^21^. Similarly, Aleksandra Sustar et al., 2019 reports lower BDNF in the CAD associated with an increased risk of cardiovascular events and mortality^22^. Additionally, BDNF showed a significant association with traditional risk factors, including diabetes, hypertension, smoking, physical activity, and obesity^23^. Similar results observed in our study relating low BDNF with a lipid profile and body mass index in CAD (Supplementary Table A1). Also, our results follows the similar outcome of Jiang H et al., 2011showing association with increased LDL, TGL, and decreased HDL levels with lower BDNF concentration in angina pectoris^24^. Interestingly, Ejiri et al., 2005 report altered BDNF in the coronary circulation between coronary sinus and aorta in patients with angina. Similarly, altered BDNF expression in human atheromatous intima, adventitia, macrophages, and smooth muscle cells in atherosclerotic coronary arteries^14^. These results suspect us to investigate the association of serum BDNF with echocardiography indices in CAD.

Multivariate regression analysis of blood parameters showed a significant contribution of neutrophils and total cholesterol with low BDNF (≤ 29.91 ng/ml) in angiogram proven CAD patients. Aleksandra Sustar et al., 2019 confirm the significant association of low BDNF with total cholesterol in coronary heart disease. Similar result was noticed in the Chinese population that relates low BDNF level with cardiovascular disease risk factors^22^. In addition to total cholesterol concentration, the increased neutrophils was noticed associated with low BDNF in CAD individuals. Neutrophils play a vital role in CAD, which promotes atherothrombotic mechanisms leading to cause myocardial infarction. Halade et al., 2013 confirm the interdependency of BDNF with neutrophils in recruiting macrophages into the infarcted region of BDNF haploinsufficient mice^25^. Besides, neutrophils involved in the release of reactive oxygen species, which activates the endothelium to deposit additional neutrophils for plaque formation. Simultaneously, our multivariate regression analysis of low BDNF group showed a significant association with echocariographic indices, including LVMI, MV E/A, and PV AR in angiogram proven CAD patients. Recently, Martin Bahls et al., 2019 demonstrated the role of BDNF on left ventricular cardiac remodeling in individuals with the risk of cardiovascular disease^17^. However, Martin Bahls et al., 2019 did not justify the role of BDNF on left ventricular dysfunction in the CAD population by comparing it with healthy control. Whereas, our results showed significant evidence of low BDNF in CAD that correlates with the echocardiograohic indices compared to healthy control. Overall, our analysis suggests low BDNF concentration is the representative of both blood parameters (neutrophils and total cholesterol) and echocardiographic indices (LVMI, MV E/A, PV AR and Biplane LVEF) in CAD.

We construct SVM models which showed significant improvement in detecting CAD with BDNF as one of the attributes (Table 5). Recently, Akella et al., 2020 use a variety of machine learning algorithms and achieved maximum accuracy of 93% in detecting CAD^26^. Interestingly our study showed benefit of adding the BDNF as one of the attribute to SVM models that represents blood parameters (neutrophils and total cholesterol) and echocardiography indices (LVMI, MV E/A, PV AR, and Biplane LVEF) in CAD (Tables 3, 4). Although our findings provides significant improvement in CAD diagnostic research, there are few limitations that to be considered before its clinical utility. First, this study includes only the South Indian population. Second, we did not follow-up on the CAD patients. Third, the changes in BDNF levels was not studied on the improvement after the treatment of CAD. Alternatively, strength of the this study need to be acknowledged that (1) selection of participants both control and CAD has been proven with coronary angiogram. (2) this study integrates the blood parameters and echocardiography indices to develop a diagnostic method that has been strengthened with a machine learning algorithm showing better accuracy in detecting CAD.

In conclusion, our study has presented a novel approach for determining the association of decreased serum BDNF with blood parameters and echocardiography indices of CAD. The machine learning (SVM) algorithm was developed to determine accuracy of BDNF with blood parameters and echocardiography indices for disease classification in CAD and healthy controls. Although all our SVM models showed better accuracy in disease classification, the model-C and model-D will be significantly improve detecting CAD using serum BDNF concentration. Therefore, our results, along with machine-based disease classification, has demonstrated the emerging evidence of BDNF in the prediction of CAD with the best accuracy value that may pave the way towards bench side clinical application.

## Methods

### Patient selection

The protocol of this study was approved by the institutional human ethical committee of the Chettinad Academy of Research and Education (IHEC/10-17/Proposal No:372). All experiments were performed in accordance with relevant guidelines and regulations. The individuals presenting angina pectoris were recruited from the Chettinad Super Specialty Hospital between October 2018 to June 2019. Written informed consent, demographic information, and health status was recorded for each participant by questionnaire. Based on the collected data, the 221 participants were selected following the exclusion and inclusion criteria. Exclusion criteria: the participants with the previous history of arterial revascularization such as stenting and bypass grafting, myocardial infarction (MI), heart failure (following the New York Heart Association Classification), valvular heart disease, pericardial diseases, idiopathic cardiomyopathy, acute or chronic infectious diseases, severe systemic disorders (malignancy, Immune diseases) and mental disorders. Inclusion criteria: (1) the participants belonging to south Indian origin between age 25 and 70 years, (2) the participants of both genders with angina, and (3) the physiological status was proven by coronary angiogram for the participant with CAD and without CAD (healthy controls, ≤ 30% lesion).

### Two-dimensional echocardiography and coronary angiogram examination

Transthoracic two-dimensional echocardiography was performed for all participants by qualified echocardiography technologist (KGM: one of the author) using the Esaote [MyLab 25Gold] following the American Society of Echocardiography guidelines. The echocardiography indices such as left ventricular (LV) mass index, biplane left ventricular ejection fraction (LVEF), early transmitral flow velocity (E), late transmitral flow velocity (A), mitral inflow E/A ratio, isovolumetric relaxation time, mitral lateral/septal peak myocardial early diastolic velocity (Le’ and Se’), pulmonary vein AR duration (PV AR), pulmonary vein systole/diastole (PV S/D) ratio, and global longitudinal strain (GLS) were calculated to assess the cardiovascular function. Simultaneously, the coronary angiogram was performed by a qualified cardiologist (CM: one of the author) using contrast agents (omnipaque and visipaque) to determine coronary artery lesions using the GE INOVA 2100-IQ PLUS with ADW DICOM. Based on angiogram report, the study participants were classified as 1) CAD patients (lesion > 30% in the primary coronary artery or its major branches) and 2) healthy controls (lesion ≤ 30%). Of those 221 participants, 116 were CAD presenting single vessel (n = 41), double vessel (n = 39), and triple vessel disease (n = 36), while the other 105 participants with proven angiogram negative were designated as healthy control.

### Quantification of serum BDNF

Overnight fasting blood (4 ml) was collected from the participants in BD vacutainer Plus Plastic Serum Tubes. The samples were immediately centrifuged at 3000 RPM for 15 min to separate serum from other cellular material and stored at − 80 °C for future analysis. Serum BDNF levels (ng/ml) were measured using the ELISA kit (R&D SYSTEMS, USA) following the manufacturer’s instructions. The BDNF concentrations were measured based on the optical density (OD) curve using known standards concentration provided within the kit. In addition, the cellular and biochemical parameters such as complete blood count, lipid profile, HbA1c, and serum creatinine were determined by standard laboratory techniques as a part of routine blood tests.

### Statistical analysis

Student t-test was performed to confirm the statistical significance of variables in CAD compared to healthy controls. Serum BDNF concentration in CAD was stratified into quartiles as low and high levels to determine its relationship with clinical and echocardiography indices. In brief, we divide CAD patients into quartiles by merging first and second quartile to have cut-off ≤ 29.91 ng/ml, represented as low BDNF group (n = 58). The third and fourth quartiles are merged obtaining a cut-off above 29.92 ng/ml, designated as high BDNF group (n = 58). Further, the multivariate regression analysis was performed to confirm the major contributing clinical and echocardiography indices associated with low serum BDNF levels in the CAD. All statistical analysis was performed using SPSS software (version 21), and the significance was considered based on *p* value < 0.05.

### Classification based on the machine learning algorithm

Support Vector Machine (SVM) is one of the efficient and widely used supervised machine learning algorithms for disease classification^27^. Prior to classification, the SVM Attribute Evaluator with ranker method^28^ was used to select most (top five) contributing blood parameter and echocardiographic indices to achieve maximum accuracy in CAD prediction. Further, we use SVM in Weka software^29^ to generate four SVM models (model-A, B, C, and D) with selected attributes from the data set (116 CAD and 105 control) for training and testing of each model. The predictor variables for model-A contains BMI, HbA1c, HDL, LDL, and Total cholesterol (Table 5). Whereas the model-B contains LVEF, LAD, L E/e, IVRT, and GLS LVEF, a top five echocardiographic indices for CAD prediction (Table 5). The attributes for model-C contains BDNF concentration as an additional attribute along with the other five predictor variables of model-A (Table 5). Similarly, the model-D contains BDNF concentration as additional attribute of model-B for CAD (Table 5). prediction. Each data set was represented with a class attribute of “CAD” or “control” for 221 instances designated based on the coronary angiogram. A tenfold cross-validation method was adopted to measure an unbiased prediction of the models. The performance of each model was assessed based on the accuracy, true positive (TP) rate, false positive (FP) rate, precision, recall, F-measure, and receiver operating characteristic (ROC). Comparing the models based on accuracy would enable us to explore the importance of BDNF in the classification of CAD from controls.

## Supplementary information

Supplementary file 1.
