# Supplementary material for: Clinical utility of brain-derived neurotrophic factor as a biomarker with left ventricular echocardiographic indices for potential diagnosis of coronary artery disease
Source: Sci Rep. 2020 Oct 1;10:16359. doi: 10.1038/s41598-020-73296-6 (PMC7530751; doi:10.1038/s41598-020-73296-6)
Supplement: Supplementary file 1 [file 41598_2020_73296_MOESM1_ESM.docx]

**Clinical utility of Brain-Derived Neurotrophic Factor as a biomarker with left ventricular echocardiographic indices for potential diagnosis of coronary artery disease**

Monisha KG ^a^, Paramasivam Prabu^b^, Chokkalingam M ^a^,Ram Murugesan ^c^, Dragan Milenkovic^d^, Shiek SSJ Ahmed ^c^*

^a^ Department of Cardiology, Chettinad Hospital and Research Institute, Chettinad Health City, Kelambakkam - 603103, Tamil Nadu, India.

^b^ School of Medicine, Department of Neurology, University of New Mexico Health Sciences Center, University of New Mexico, USA

^c^ Drug Discovery and multi-omics Laboratory, Faculty of Allied Health Sciences, Chettinad Academy of Research and Education, Kelambakkam - 603103, Tamil Nadu, India

^d^ Université Clermont Auvergne, INRAe, UNH, Clermont-Ferrand, France

Table A1. Serum BDNF quartile levels in CAD categorized by associated risk factors. Patients were grouped into two as low BDNF (≤29.92 ng/ml) and high BDNF (>29.92 ng/ml) and values represented with average± standard deviation.

| **CLINICAL PARAMETERS** | **LOW BDNF** | **HIGH BDNF** | ***p* value** |
| --- | --- | --- | --- |
| Age (years) | 57±10.76 | 57.89±10.2 | 0.507 |
| BMI (kg/m2) | 25±3.064 | 27.36±2.61 | **< 0.001** |
| Systolic BP (mmHg) | 119±17.37 | 128.43±16.2 | **0.003** |
| Diastolic BP (mmHg) | 80±13.87 | 86.29±11.7 | **0.006** |
| Platelet (LAC/C.mm) | 3.5±0.996 | 2.30±0.71 | **< 0.001** |
| Basophil (%) | 1.2±0.737 | 0.61±0.44 | **< 0.001** |
| Eosinophil (%) | 7.9±2.714 | 5.16±2.95 | **< 0.001** |
| Lymphocyte (%) | 43±16.41 | 29.97±8.91 | **< 0.001** |
| Monocyte (%) | 9.1±2.578 | 6.63±1.97 | **< 0.001** |
| Neutrophil (%) | 65.69±17.95 | 50.09±13.7 | **< 0.001** |
| HbA1c (%) | 7.1±1.182 | 5.74±0.61 | **< 0.001** |
| HDL cholesterol (mg/dL) | 33±7.491 | 38.5±8.14 | **< 0.001** |
| LDL cholesterol (mg/dL) | 154±33.55 | 111.97±727.3 | **< 0.001** |
| TGL cholesterol (mg/dL) | 173±62.95 | 111.19±33.5 | **< 0.001** |
| T.Cholesterol (mg/dL) | 223±72.19 | 166.14±43.9 | **< 0.001** |
| Serum Creatinine (mg/dL) | 1.3±0.347 | 0.83±0.31 | **< 0.001** |

Bold values indicate significance at *p* <0.05.

Table A2. An Association of BDNF levels stratified with the echocardiographic parameters in CAD patients. Echocardiographic data of low and high BDNF group in CAD (≤ 29.91 ng/ml and >29.92 ng/ml) with average±standard deviation.

| **ECHO INDICES** | **LOW BDNF** | **HIGH BDNF** | ***p value*** |
| --- | --- | --- | --- |
| Biplane LVEF (%) | 49.48±5.59 | 40.01±4.741 | **< 0.001** |
| LAD (cm) | 3.52±0.44 | 3.38±0.582 | 0.129 |
| LVMI (g/m2) | 90.89±20.91 | 110.5±23.754 | **< 0.001** |
| MV E/A | 0.50±0.51 | 0.78±0.35 | **0.001** |
| S E/e' | 7.27±1.44 | 7.14±1.106 | 0.589 |
| L E/e' | 7.93±1.00 | 7.31±1.195 | **0.003** |
| IVRT (ms) | 100.82±18.38 | 128.32±20.063 | **< 0.001** |
| PV AR (m/s) | 29.15±5.44 | 26.41±4.683 | **0.004** |
| PV S/D | 0.96±0.35 | 1.63±0.57 | **< 0.001** |
| GLS EF (%) | -14.77±1.04 | -13.11±0.88 | **< 0.001** |

Bold values indicate significance at *p* <0.05.
